# Where Are We Now with Oncolytic Viruses in Melanoma and Nonmelanoma Skin Malignancies?

**DOI:** 10.3390/ph17070916

**Published:** 2024-07-09

**Authors:** George Nassief, Angela Anaeme, Karen Moussa, David Chen, George Ansstas

**Affiliations:** 1 Division of Medical Oncology, Department of Medicine, Washington University in Saint Louis, Saint Louis, MO 63110, USA; 2 UMKC School of Medicine, University of Missouri Kansas City, Kansas City, MO 64108, USA; 3 Division of Dermatology, Department of Medicine, Washington University in Saint Louis, Saint Louis, MO 63110, USA

**Keywords:** oncolytic viruses, melanoma, squamous cell carcinoma, basal cell carcinoma, Merkel cell carcinoma, T-VEC, RP-1

## Abstract

Skin cancer prognosis has greatly improved recently due to the introduction of immune checkpoint inhibitors (ICIs). However, many patients with advanced skin cancer still experience immunotherapy resistance and disease progression during ICI treatment, thus calling for novel therapeutics which address this treatment gap. Talimogene laherparepvec (T-VEC) has gained popularity in recent years as a viable treatment option for patients with skin cancer. In preclinical studies, T-VEC demonstrated both a direct anti-tumor effect in injected lesions as well as a systemic immune-mediated effect in non-injected lesions, which could pose additional benefits when combined with ICI therapy. Following promising results from the OPTiM trial, the Food and Drug Administration (FDA) approved the usage of T-VEC as a single agent in advanced melanoma. However, the MASTERKEY-265 trial demonstrated that adding T-VEC to pembrolizumab did not offer additional clinical benefit in patients with melanoma. Nevertheless, the promising efficacy of T-VEC and its approval by the FDA helped oncolytic viruses (OVs) gain wide attention in cancer therapy, and extensive research has been undertaken to evaluate the usage of OVs in other tumors such as sarcomas and breast cancers. Here, we provide a review of clinical results from 2022 to 2024 that investigate the efficacy and safety of OVs as a monotherapy or in combination with other therapies in skin malignancies. Furthermore, we delineate the current limitations in OV utilization and outline future directions to enhance clinical outcomes for patients with skin malignancies receiving OV-based therapies.

## 1. Introduction

Skin cancer is the most common cancer in the United States [1,2]. It is estimated that nonmelanoma skin cancers affect more than 3 million Americans yearly [3,4,5]. In addition, the American Cancer Society predicted over 100,000 new cases and 8000 new deaths from melanoma alone in 2024 [6]. Although invasive melanoma only accounts for 1% of skin cancers diagnoses, the disease makes up the majority of deaths [7]. Basal and squamous cell carcinomas, the most common skin cancers, are highly treatable if detected early, with estimates of the 5-year relative survival rates being higher than 95% [7,8,9]. Overall, the relative 5-year survival rate for melanoma is 94% [7]. However, distant-stage disease has a 5-year survival rate of 32% [7].

Advancements in treatment like ICIs have notably decreased the mortality of melanoma, especially in advanced disease. The Checkmate-067 trial estimated a 7.5-year survival rate of 48% in all patients with advanced melanoma treated with the combination of ipilimumab–nivolumab [10]. However, many patients with melanoma still experience therapy resistance, intolerable side effects, and disease progression following an initial response to immunotherapy treatment [11,12,13,14]. This calls for novel therapeutics that address this gap in treatment options, especially for patients with advanced disease.

In 2015, the FDA approved T-VEC as the first oncolytic herpes simplex virus type 1 (HSV-1) for recurrent melanoma treatment following surgery [15,16]. Following this, OVs have gained wide attention as a therapeutic option for patients with cancer experiencing disease progression while undergoing standard therapy. OVs work by infecting and replicating rapidly in cancer cells, resulting in cell lysis and immune system exposure to intracellular tumor antigens. In addition, OVs could carry other genes into cancer cells to boost their tumor destruction ability. OVs can also stimulate the immune system against cancer cells, thereby enhancing the anti-tumor response in injected and non-injected lesions [17].

OVs are primarily administered through intratumoral (IT) injections. Upon injection, OVs replicate in tumor cells, leading to the oncolysis of these cells. This turns the tumor microenvironment (TME) into an inflammatory site, thereby recruiting many immune cells such as T lymphocytes, which work to rid the TME from cancer cells. Furthermore, this immune-stimulatory response against tumor antigens enhances immunological memory against cancer cells [18]. OVs present with a major therapeutic advantage over other therapeutic routes since they are administered locally, which reduces exposure to systemic side effects. Additionally, OVs only target cancer cells, leading to oncolysis without infecting healthy tissue, giving them a major advantage compared to other therapeutic approaches [19].

Importantly, many studies have shown benefits preclinically and clinically in combining ICI and OV therapy in different cancers, including melanoma [20]. Indeed, OVs have demonstrated the ability to reverse unresponsive TMEs, turning them from “cold” to “hot”, which are characterized by a low and high quantity of immune cells, respectively, therefore making tumor cells more susceptible to ICI therapy [21]. For instance, the combination of OVs and T-cell therapies has been shown to address the inherent limitation that each treatment agent brings in solid tumors [22], thereby raising interest in the combination of OVs and immunotherapy. However, OV response varies greatly among different patients, as they are dependent on the host’s immune response, treatment timepoint, type of malignancy, and the type of OVs administered [23]. Thus, extensive research is still needed for patient selection for OV therapies as well as for a better understanding of how to administer the treatment in accordance with standard-of-care therapeutics.

Currently, many clinical studies are underway to investigate the efficacy and safety of novel OVs as a monotherapy or in combination with other therapeutic options in patients with skin cancers. Ziogas et al. provided an extensive literature review on preclinical and clinical studies that evaluated the efficacy and safety of OVs in skin cancers up until 2022 [23]. In this review, we aim to provide an update on the status of major clinical trials and outcome data from 2022 to April 2024 by querying ClinicalTrials.gov to expand upon current knowledge of the potency and safety profile of OVs in skin cancers. We also address the limitations that are present in the field as well as the future directions regarding how OVs could be implemented in skin cancer treatment.

## 2. Current State of OVs in Melanoma

Herpes simplex viruses (HSVs) have presented major therapeutic advantages in OV therapy due to their selective viral replication, low toxicity, and the presence of effective anti-herpetic medications in case of uncontrolled spreading [23]. In addition, HSV-1 has four surface-bound nectin-1 receptors, allowing for ease of access for infection and replication in all tumor cell types. This multi-receptor property allows for rapid tumor cell killing, making them ideal OV therapies compared to other viruses, as they are less prone to resistance [15,24]. T-VEC is a modified HSV-1 virus with the deletion of the ICP34.5 and ICP47 genes. This OV is genetically modified to decrease the pathogenicity of the virus and selectively target tumor cells. This oncolytic virus also expresses the granulocyte-macrophage colony-stimulating factor (GM-CSF), which recruits dendritic cells and promotes them from mature to antigen-presenting cells [25].

Following T-VEC, RP-1 was developed as a modified version of the original HSV-1-based oncolytic virus [23]. Similar to T-VEC, RP-1 is a genetically engineered HSV-1 virus that targets tumor cells and expresses GM-CSF. Unlike T-VEC, RP-1 has a more potent anti-tumor cytotoxic effect and expresses a fusogenic protein, GALV-GP-R, which has been found to preclinically promote immunogenic cell death. In addition, RP-1 can express anti-CTLA-4 or other immune co-stimulatory pathway-activating ligands to increase anti-tumor activity by enhancing the systemic immune response against tumor cells [26].

**Figure 1:**
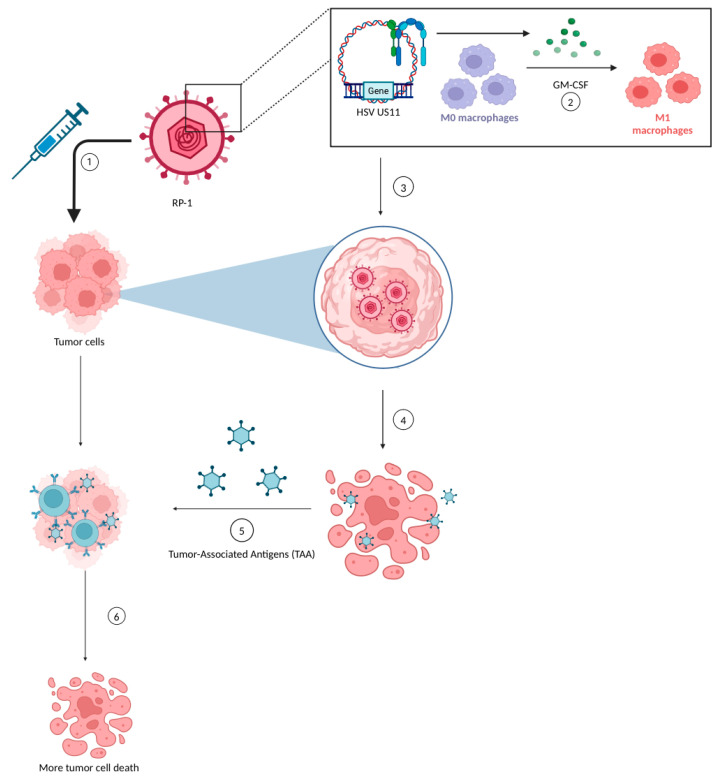
Mechanism of RP-1 [Figure 1]: (1) The RP-1 oncolytic virus is injected into the tumor cells. (2) Viral DNA replaces the tumor DNA and secretes GM-CSF that causes the recruitment of macrophages and other leukocytes. (3) The HSV US11 gene promotes viral DNA replication inside the tumor cells. (4) Viruses cause the oncolysis of tumor cells, which releases tumor-associated antigens (TAAs). (5) TAAs further infiltrate other tumor cells and promote a cytotoxic T-cell response. (6) Further tumor cell death is induced through apoptosis. Created with BioRender.com.

RP-1 works through a mechanism known as gene-mediated cytotoxic immunotherapy, which involves delivering viral DNA which would replace the tumor cells’ DNA, producing GM-CSF [27]. The GM-CSF acts as a recruiter for leukocytes to the tumor sites to target and destroy only neoplastic cells [28]. Similar to T-VEC, RP-1 also contains ICP34.5 and ICP47 gene deletions that permit viral replication in tumor cells by upregulating the HSV US11 gene. The HSV US11 gene promotes viral replication in tumor cells with selectivity directed only towards cancerous cells, sparing healthy cells in the body [23]. These viruses eventually lyse the tumor cells, thereby killing these cells and releasing tumor-associated antigens (TAAs) which exert an anti-tumor response, leading to more tumor cell death. A unique feature of this therapy is that the RP-1 virus itself can trigger a cytotoxic T-lymphocyte (CTL) response, which can potentially induce apoptosis in nearby non-infected tumor cells [28] (Figure 1).

Multiple other virus vectors, such as the adenovirus and the poliovirus, have also been evaluated in recent years as viable OV-based therapies in melanoma. Here, we present updates on the results of clinical trials that investigated the effectiveness and safety profile of OV therapies in melanoma from the 2022 to 2024.

### 2.1. Current State of T-VEC Usage in Melanoma

Preclinically, T-VEC demonstrated a high efficacy in injected tumors and an abscopal effect in non-injected tumors [16,29,30]. In addition, OncoVEXmGM-CSF (T-VEC with mouse GM-CSF) demonstrated preclinical efficacy in increasing tumor-specific CD8+ T-cell and systemic efficacy in tumor cells when combined with a checkpoint blockade in murine mouse models [31,32]. These studies provided a framework for studying the clinical efficacy of T-VEC as a monotherapy or in combination with ICI treatment.

Following positive results in early phase I (NCT03064763) and II (NCT00289016) clinical trials testing the efficacy and safety of T-VEC in melanoma, a phase III (OPTiM trial) randomized open-label trial (NCT00769704) was initiated, testing T-VEC against subcutaneous GM-CSF in unresectable stage IIIB–IVM1c melanoma. The study had a median follow-up of 49 months. At that follow-up, the study observed an overall survival (OS) of 23.3 and 18.9 months for the T-VEC and GM-CSF treatment groups, respectively (HR = 0.79; *p* = 0.0494). The objective response rate (ORR) was reported to be 31.5%, with a 16.9% complete response (CR), and 6.4% with a 0.7% CR, respectively, for the T-VEC and GM-CSF arms. Furthermore, the durable response rate (DRR), which is the percentage of patients demonstrating a complete or partial response for a minimum of 6 months, was reported as 19.0% and 1.4% (*p* < 0.0001), respectively, for the T-VEC and GM-CSF arms. In addition, an 88.5% survival rate at the 5-year landmark analysis was observed in patients with CR [33]. In the study, biomarker analysis demonstrated that T-VEC increases effector CD8+ T-cells and NK cell infiltrates in non-injected lesions, which indicates a systemic effect that could be further extrapolated by combining the treatment with immunotherapy [34]. With these promising results, the FDA approved the usage of T-VEC in advanced melanoma. Importantly, the systemic immune-mediated anti-tumor effect highlighted the need to investigate the potential synergic efficacy of combining ICI with T-VEC.

The combination of pembrolizumab and T-VEC vs. pembrolizumab alone was then tested in the largest phase III trial, MASTERKEY-265, in stage IIIb/IVM1c melanoma (NCT02263508). In the study, the median progression-free survival (PFS) was 14.3 months and 8.5 months for the T-VEC and pembrolizumab combination therapy and pembrolizumab with placebo (HR 0.86; *p* = 0.13), respectively. In addition, the ORR was 48.6% for the combination therapy, with a CRR of 17.9%, and 41.3% for pembrolizumab and the placebo, with a CRR of 11.6%. The DRR was 42.2% in the combination therapy and 34.1% in the pembrolizumab and placebo group. The final results of the study found no significant difference in the combination treatment vs. pembrolizumab alone in improving the PFS or the OS [35]. Interestingly, the PFS was better in the combination therapy in three patient subgroups, including those enrolled in the United States. The study speculates that this regional difference could be attributed to patients enrolling in the US having LDH ≤ the upper limit of normal and/or with the sum of the longest diameters of target lesions ≤ the median, which were the other two subgroups that had the PFS advantage [36]. Nonetheless, more research is warranted to better understand the regional differences in PFS among the different treatment arms. More importantly, there is still a need for future studies to investigate how T-VEC could effectively be combined with ICI to improve patient outcomes.

Recently, the MASTERKEY-115 trial gave its final results at the ASCO 2022 conference, where it tested the efficacy and safety of T-VEC and pembrolizumab in patients with unresectable or metastatic stage IIIB–IVM1d melanoma who progressed on anti-PD-1 therapy (NCT04068181). In the study, both cohorts 1 and 2 were patients administered anti-PD-1 in a recurrent or metastatic setting who had progressed on anti-PD-1 within 12 weeks of the final dosage. Cohort 1 was segmented by those having primary resistance, while cohort 2 patients had acquired resistance. Cohorts 3 and 4 both received anti-PD-1 in the adjuvant setting and were disease-free for less than 6 months or more than/equal to 6 months, respectively, before progressing with therapy. The study found an ORR of 0% and 6.7% in cohorts 1 and 2, respectively. In addition, it found an ORR of 40% and 46.7%, respectively, for cohorts 3 and 4. The most reported TRAEs in the study were pyrexia, fatigue, and influenza-like symptoms. In addition, the study reported 12.7% of patients experiencing grade 3 or higher TRAEs [37] (Table 1). Thus, these results demonstrated promise in this combinatorial approach following anti-PD-1 failure in melanoma in certain clinical scenarios, particularly those in which anti-PD-1 failure occurs in the adjuvant setting. However, more research is warranted to translate these results in larger clinical trials of patients with melanoma experiencing disease progression while undergoing ICI treatment to better understand the potential benefit behind this combinatory approach.

Another phase I study (NCT03064763), which was recently completed in early 2023, investigated the safety and efficacy of T-VEC in Japanese patients with unresectable stage IIIB–IVM1c melanoma. The study found that 77.8% of patients had TRAEs. The most common TRAEs reported were pyrexia, malaise, decreased appetite, pruritus, and skin ulcers. In the study, 11.1% of patients had a durable partial response of more than or equal to 6 months (Table 1). The study reported a similar safety and efficacy profile of the combinatory approach to the OPTiM trial (NCT00769704) [42]. Since the efficacy and safety reports of treatments evaluated in clinical trials do not often include the results in melanoma subtypes like acral lentiginous melanoma, and due to the differences in people’s response to treatment in melanoma subtypes, it is important to investigate the outcomes following a treatment approach in different patient cohorts, like this study. Interestingly, recent statistical reports demonstrated that acral lentiginous melanoma was prevalent in 41% of patients with melanoma in Japan [48]. However, in this study, superficial spreading melanoma and nodular melanoma, followed by acral lentiginous melanoma, were the most common subtypes [42]. These are not typical of Japanese patients, which could explain the similarity in outcomes to the OPTiM trial (NCT00769704). Therefore, more research is required to understand the efficacy of this treatment option in larger Japanese patient cohorts as well as in other Asian patient cohorts.

In addition, two phase II trials have presented recent results that tested the efficacy of the combination of T-VEC with other agents as a neoadjuvant therapy. The final 5-year analysis of the largest trial to evaluate T-VEC in the neoadjuvant setting was recently published (NCT02211131). In this trial, patients with resectable stage IIIB to IVM1a melanoma were segmented into two groups, with group 1 given six neoadjuvant T-VEC doses preceding surgery and group 2 only undergoing a prompt surgery. The analysis performed on both groups demonstrated that group 1 had a recurrence-free survival (RFS) of 22.3%, while group 2 had an RFS of 15.2% (HR: 0.76). In addition, the study found an event-free survival (EFS) of 43.7% in group 1 and 27.4% in group 2. The overall survival was 77.3% in group 1 and 62.7% in group 2 (HR: 0.54). The study demonstrated the HRs for local RFS to be 0.82, the regional RFS to be 0.81, and distant metastasis-free survival to be 0.73 [43] (Table 1). In addition, in the two-year interim analysis, increased CD8+ density correlated with the clinical outcomes [49]. These results hold great promise as they demonstrate a durable response in utilizing T-VEC in the neoadjuvant setting. This is greatly important in understanding how T-VEC could fit into the treatment algorithm against metastatic disease. It is, thus, imperative to further evaluate these findings in phase III clinical trials.

Similarly, the NIVEC trial, a phase II trial (NCT04330430), had recently evaluated the usage of T-VEC and nivolumab in the neoadjuvant setting. The treatment consisted of four intralesional T-VEC and three nivolumab 240 mg doses given every two weeks prior to surgery. The study demonstrated a pathological response rate of 74% with a major pathological response (MPR, complete pathologic response + near-complete response with fewer than 10% viable tumor cells) in 65% of patients and a partial response in 9% of patients. A total of 8% of patients experienced grade 3 TRAEs in the study, and the 1-year EFS was 75% [38] (Table 1). These results again make a strong case for utilizing T-VEC in the neoadjuvant setting in advanced melanoma; however, more research is warranted to expand these results in phase III studies.

In addition, two studies recently presented the results of combining T-VEC with other therapeutic arms. In a phase I study, the usage of IT injection of T-VEC and CD1c (BDCA-1)+ myeloid dendritic cells (myDCs) as well as CD141 (BDCA-3)+ myDCs in advanced melanoma patients who did not respond to standard therapy was evaluated (NCT03747744). T-VEC was administered on day 1, followed by CD1c (BDCA-1)+ myDCs +/− CD141 (BDCA-3)+ myDCs on day 2. T-VEC injection was repeated on day 21 and every 14 days thereafter. In three sequential cohorts 1–3, the number of administered D1c (BDCA-1)+ myDCs was increased from 0.5 × 10^6^ to 1 × 10^6^ to 10 × 10^6^ cells, respectively. Cohort 4 received all isolated CD1c (BDCA-1)+/CD141 (BDCA-3)+ for injection. The results demonstrated a safe profile, with the most frequent adverse events being fatigue, fever, and influenza-like symptoms. Two out of the three patients in cohort 3 experienced a durable, pathologically confirmed complete response that was ongoing 33 and 35 months after the study’s start date. One patient in cohort 4 (*n* = 6) had an unconfirmed partial response, and two patients had a mixed response. The on-treatment biopsies in this study demonstrated strong inflammatory cell infiltration in regressing lesions [39] (Table 1). Thus, these results highlight a tolerable and promising anti-tumor activity in ICI-refractory melanoma when combining CD1c (BDCA-1)+ +/− CD141 (BDCA-3)+ myDCs with T-VEC. Importantly, with the increased focus on cell therapies in melanoma, these results underscore the value of further investigating the potential synergism that may exist in utilizing T-VEC with other cell therapies similar to myDCs.

Furthermore, another phase II clinical trial (NCT02819843) recently presented the outcomes of combining T-VEC with radiotherapy or not in patients with cutaneous metastasis from solid tumors. One patient in the combination treatment and one patient in the T-VEC single-arm treatment demonstrated a CR in the largest non-irradiated and non-injected metastasis but progressed in new metastases. The composite response rate in non-injected and irradiated tumors was 13% and 0%, respectively, in T-VEC alone and the combination therapy, respectively. The composite response rate in the treated tumors was 7% in the single-arm T-VEC treatment and 22% in the combination treatment. However, due to no patients exhibiting overall modified responses with respect to the World Health Organization and immune-related response criteria and the trial’s slow accrual, this trial was closed after the first enrollment stage. Grade 3–4 TRAEs occurred in two patients in each arm, with similar frequencies. The most common adverse event was fever [41] (Table 1). The results in this trial demonstrate a higher response in the combination approach, but a more frequent response in non-injected or irradiated tumors in the single-arm T-VEC group. Thus, the mixed results of this study underscore the need for future research testing the safety and effectiveness of this combinatory approach in cutaneous malignancies.

### 2.2. Current State of RP-1 Therapy

As mentioned, RP-1 is derived from a different HSV-1 strain than T-VEC, which is selected for an increased cytotoxic effect against cancer cells [26]. When tested in mouse lymphoma and human lung and breast cancer cells, RP-1 demonstrated increased efficacy when combined with anti-PD-1 therapy [26]. This led the way for RP-1 to be tested in multiple clinical trials in solid tumors, including melanoma, in combination with ICI.

The IGNYTE trial (NCT03767348), a phase II clinical trial, recently published results evaluating the efficacy of combining RP-1 with nivolumab in patients with advanced melanoma who did not respond to anti-PD-1 therapy. The ORR from the trial was reported as 37.4%, with 18.7% of patients achieving a CR. The trial also noted that non-injected lesions showed a response in most of the responding patients. A total of 85.3% of the responses were ongoing after 3.7–36.6 months from therapy administration. The most common TRAEs were fatigue, chills, pyrexia, and nausea, with most of them being grade 1–2 [40] (Table 1). These clinical findings demonstrate a significant anti-tumor response when combining RP-1 and nivolumab, which is durable following anti-PD-1 failure. Therefore, there is a need to expand these findings in phase III clinical studies due to the promising results demonstrated. This is especially important given the limited therapeutic options available following standard immunotherapy failure in patients with advanced melanoma.

### 2.3. Advancements in Other OV Therapies

OrienX010, another HSV-1-derived OV which expresses GM-CSF, was tested in advanced melanoma, and recent results were published, highlighting the efficacy and safety of the therapy in resectable stage IIIb-IV (M1a) acral melanoma in a phase IB clinical trial (NCT04197882). In this trial, OrienX010 and toripalimab were given as neoadjuvant therapy for four to six weeks prior to resection, followed by adjuvant toripalimab for up to a year. The study observed a radiological ORR of 36.7%, with a radiological CRR of 3.3%, and a pathological ORR of 77.8% with a 14.8% pathological CRR. A 1-year RFS of 80.0% was observed in the study. In addition, 17% developed grade 3 adverse events, which included soft tissue infections, transaminitis, peripheral neuropathy, and neutropenia. No grade 4 adverse events were reported [44] (Table 1). These findings are especially important given the limited treatment options for acral melanoma and its worse prognosis compared to other cutaneous malignancies. More importantly, these promising results highlight the need for more investigation into the efficacy and safety of HSV-1-based OVs in melanoma sub-cohorts such as acral and uveal melanoma, which have a worse prognosis than cutaneous melanoma.

Another study in solid tumors, including melanoma and head and neck squamous cell carcinoma (HNSCC), evaluated the potency and safety profile of T3011 (NCT05602792). Briefly, T3011 is a genetically modified oncolytic HSV, with the deletion of one copy of ICP34.5 and the retention of ICP47 to maximize virulence attenuation and replication activity. It has also been modified with inserted IL-12 and anti-PD-1 antibody to improve the TME’s responsiveness to immunotherapy and elicit specific anti-tumor immunity.

In a phase I/IIa clinical trial, T3011 was administered via IT injections as a single agent in a dose escalation study. Patients who progressed or developed intolerable side effects to standard therapeutics received T3011 IT therapy Q2W in several dose cohorts, ranging from 2.5 × 10^5^ to 1 × 10^8^ PFU/mL. With a median follow-up of 5.7 months, the study highlighted 2.2% of patients experiencing grade 3 or higher TRAEs, with the most common being pyrexia, influenza-like symptoms, TSH increase, proteinuria, facial edema, white blood cell count decrease, AST increase, and anemia. RP2D was determined to be 1 × 10^8^ PFU/mL. A total of 55 pts, who were under RP2D and could be evaluated for their tumor response, demonstrated an ORR of 11% and a DCR of 49%. In all the 76 patients who received T3011 under RP2D, the median PFS was 2.8 months (Table 1). Overall, T3011 monotherapy demonstrated encouraging anti-tumor activity without the possibility of transmission to local and systemic environments [45]. Furthermore, the treatment demonstrated significant efficacy in HNSCC (Table 2), as highlighted below, which underlines the importance of future investigation of T3011 in phase II studies in skin cancer cohorts.

Due to their immunogenic nature, ease of production, and tolerance in human subjects, adenoviral agents have also been studied in recent years as a treatment option in skin cancers [23]. TILT-123 was first introduced in 2021 as an oncolytic adenovirus that expresses two cytokines, TNFa and IL-2, promoting T-cell stimulation in the TME. The treatment was tested in rodents and demonstrated good tolerance as a monotherapy or in combination with other ICI [50]. This allowed for the initiation of a phase I clinical trial that tested TILT-123 with tumor-infiltrating lymphocytes (TILs) in patients with advanced melanoma (NCT04217473). In that trial, patients with stage IV melanoma were treated with multiple TILT-123 and one or two injections of TILs, which were grown from resected tumor tissue. Imaging on day 78 showed a RECIST1.1 response in two patients, and the study observed a disease control rate (DCR) of 38%. Among these two responding patients, one had an ongoing partial response, while the other had a durable complete response. In addition, long-lasting stable disease was reported in two patients for more than 10 months. PET evaluation was also performed, and a DCR of 46.1% was reported based on the patients who could be evaluated, with four patients having a partial or minor response. Following four TILT-123 injections and before TILs treatment, four partial or minor responses were also observed based on the PET scans (Table 1). Thus, these preliminary results demonstrate the safety profile of this combination therapy as well as the demonstrated clinical activity in patients with advanced disease [47]. Furthermore, this study highlights the potential promise that adenoviral oncolytic viruses hold in the treatment of patients with advanced melanoma.

**Table 2:** Basal, squamous, and Merkel cell recent clinical trials and outcomes.

Authors, Study Name, NCT #	Trial Stage	Therapeutic Approach	N	Disease/Stage	Outcomes	Common TRAE/Grade 3–4
Ramelyte et al., 2023 (NCT03458117) [51]	Phase I (Completed)	T-VEC	27	Locally advanced SCC, BCC, MCC, and cutaneous T-cell lymphoma	84% response in injected tumors, 40% non-injected response, 84% reduction in elevation, and 68% reduction in redness.	The most common TRAEs experienced were fever, flu-like symptoms, and the ulceration of the injected lesion.
(NCT04163952)	Phase I (Active, Not Recruiting)	T-VEC + Panitumumab	5	SCC	N/A	N/A
Haydon et al., 2022, CERPASS (NCT04050436) [52]	Phase II (Active, Not Recruiting)	RP-1 + Cemiplimab vs. Cemiplimab	231	SCC	There was a 48.1% CRR in RP1 + cemiplimab vs. 22.6% in cemiplimab alone. There were comparable ORRs between the combination (52.5%) and single-therapy groups (51.4%).	The RP1-receiving group experienced flu-like symptoms. The non-RP1-receiving group experienced fatigue, pyrexia, pruritis, nausea, and chills.
Curiel-Lewandrowski et al., 2024 (NCT03714828) [53]	Phase II (Completed)	T-VEC	11	SCC	ORR = 100%, mDoR = 209 days, Time to response = 35 days. In injected lesions, 95.8% CRR, and 4.2% partial response.	Most common TRAEs = fatigue, flu-like symptoms, and headache; all TRAEs were grade 1–2.
Migden et al., 2024, ARTACUS (NCT04349436) [54]	Phase Ιb (Recruiting)	RP-1	65	SCC, BCC, MCC	Interim results: ORR = 35%, with a CRR of 22%.	Most common TRAEs were fatigue, chills, and pyrexia.
(NCT03921073)	Phase II (Terminated)	T-VEC	5	Angiosarcoma of the skin	N/A	Most common TRAEs were fever, flu-like symptoms, fatigue, and injection site pain.
Kelly, C. et al., 2023 (NCT03069378) [55]	Phase II (Active, Not Recruiting)	T-VEC + pembrolizumab	21	Locally advanced/metastatic sarcoma	The best ORR by 24 weeks was 11% for undifferentiated pleomorphic sarcoma and myxofibrosarcoma (UPS/MFS), 71% for cutaneous angiosarcoma (AS), and 0% for epithelioid sarcoma (ES). The mPFS was 14.9 for UPS/MFS and 54 for AS.	Only one participant in the AS expansion cohort experienced a grade 3 TRAE (immune-mediated hepatitis); no participants experienced grade 4 TRAEs.
Ji et al., 2023 (NCT05602792) [45]	Phase I/IIa (Recruiting)	T3011	233	Advanced solid tumors	The confirmed ORR was 11%; the DCR was 49% in 55 pts, including patients with HNSCC, sarcoma, melanoma, breast cancer, etc., treated under RP2D who could be evaluated for their tumor response. The median PFS was 2.8 months in all 76 pts receiving T3011.	Both TRAEs of ≥G3 and treatment discontinuation in 2.2% of pts. The most frequently reported TRAEs were pyrexia (21.1%), influenza-like illness (8.9%), increased TSH (6.7%), proteinuria (6.7%), facial edema, white blood cell count decreased, AST increased, and anemia (5.6%).

Abbreviations: N/A = not available; N = number of patients; ORR = objective response rate; DoR = duration of response; PFS = progression free survival; TRAEs = treatment-related adverse events; T-VEC = talimogene laherparepvec; SCC = squamous cell carcinoma; BCC = basal cell carcinoma; MCC = Merkel cell carcinoma; and HNSCC = head and neck SCC.

## 3. Current State of OVs in NMSCs

Recent research on the utility of OVs in treating metastatic disease has led to great strides in the improvement of treatment options for those with advanced melanoma. However, there is a scarcity of completed studies that demonstrate the same efficacy of T-VEC for nonmelanoma skin cancers (NMSCs) [56]. Given that T-VEC was initially approved by the FDA for usage only in melanoma and much of the current literature focuses on its efficacy in treating melanoma tumors, it is difficult to extrapolate the results from such studies to NMSCs. Additionally, given that surgical removal is typically the first-line therapy and curative for most NMSCs, research on the medical treatment options for nonmelanoma has been historically lacking compared to melanoma [57]. Nevertheless, several studies seeking to demonstrate the effectiveness of T-VEC in NMSCs are currently in progress to fill this knowledge gap.

In this section, we provide pertinent updates on clinical trials investigating OV therapy for nonmelanoma skin cancers. The treatment of nonmelanoma skin cancers requires special consideration, as this population faces unique barriers and challenges in treatment which patients with metastatic melanoma may not. Specifically, nonmelanoma skin cancer is the most common malignancy among recipients of solid organ transplants, placing this population at a higher risk and complicating the treatment of these cancers with traditional systemic immunotherapies, as the side effects often outweigh the benefits [58]. Given these special considerations, the investigation of OVs as a treatment option for NMSCs is of great importance.

Below is a summary of the most recent results from clinical trials from 2022 to April 2024 that investigated OVs in nonmelanoma skin cancers.

### 3.1. T-VEC Usage in NMSCs

Given its efficacy in improving the outcomes for advanced and metastatic melanoma, T-VEC is being investigated as a monotherapy option for NMSCs. Ongoing and recently completed trials are yielding very encouraging results. In a recently completed phase II trial (NCT03714828), the usage of T-VEC as a monotherapy for cutaneous SCC was evaluated. The results were incredibly promising, with 100% of patients achieving an overall response, 90.9% demonstrating a CR, and 9.1% demonstrating a partial response. Of the 24 lesions that were injected during the study, 100% achieved an overall response, 95.8% achieved a CR, and 4.2% achieved a partial response. Further, the median time to response was 35 days (18–122), and the median duration of the response was 209 days (136–250). In terms of TRAEs, the patients most often experienced fatigue, flu-like symptoms, and headache. In comparison to an interim data analysis conducted in 2022, both the mean time to response and the mean duration of the response improved, and the patients presented with fewer invasive tumors than they had 1 year prior to beginning T-VEC therapy (*p* = 0.0156) and 2 years prior to T-VEC therapy (*p* = 0.0312) [59]. The response rate highlighted by this study along with the overall safety profile highlight the value of using T-VEC in NMSC treatment. It is, however, important to expand these results in phase III trials investigating T-VEC in this patient cohort.

Another trial completed in 2023 (NCT03458117) found similarly promising results when investigating T-VEC monotherapy for cutaneous lymphomas and advanced NMSCs. Among the 27 participants, 19 had cutaneous B cell lymphoma, 5 had cutaneous T-cell lymphoma, 1 had cutaneous SCC, and 1 had cutaneous Merkel cell carcinoma (MCC). Of the injected lesions, 84% demonstrated a response, with a reduction in elevation of 84%, a reduction in redness of 68%, and a non-injected response of 40%. The overall response rate was 32%. Regarding safety, 34% experienced TRAEs, and the most common were fever, flu-like symptoms, and ulceration of the injected tumor. Three patients (24%) experienced grade 3 TRAEs [51]. This study suggests not only a significant response to T-VEC monotherapy among the injected tumors but also an abscopal response, demonstrating systemic effectiveness against NMSCs. The results of these studies are favorable for the future of T-VEC monotherapy in NMSC treatment, and they suggest the great utility of OV therapy in future trials. In addition, the abscopal effect demonstrated highlights the potential value that may exist in combining T-VEC with immunotherapies.

Currently, several well-established immunotherapies are approved for use in NMSCs [60]. Specifically, cemiplimab, pembrolizumab, and avelumab are FDA-approved for use in metastatic MCC and SCC and have demonstrated promising early results for the future of NMSC treatment. However, there is great interest in combining these therapies with T-VEC and other OVs, given the increased success in the response rates achieved with these combination therapies in melanoma. A previously published phase I trial (NCT02626000) investigating T-VEC and pembrolizumab combination therapy in metastatic HNSCC found mildly encouraging results: the confirmed ORR was found to be 16.7% (95% CI, 6.4–32.8); the stable disease rate was 22.2% (*n* = 8); the progressive disease rate was 16.7% (*n* = 6); the DCR was 38.9% (95% CI, 23.1–56.5); the median PFS was 3.0 months (95% CI, 2.0–5.8); and the OS was 5.8 months (95% CI, 2.9–11.4) [61]. Despite these promising findings, the investigators decided not to proceed with the phase III arm of the study, warranting further exploration of the efficacy and reproducibility of these preliminary results. Thus, more research is warranted in exploring the possible synergy that may exist between standard immunotherapy and T-VEC in NMSCs. Importantly, further research is still needed to understand how T-VEC could fit in the therapeutic algorithm against NMSCs as a monotherapy or in combination with other therapies.

More recently, an ongoing phase II trial (NCT03069378) has been investigating how effective the combination therapy of T-VEC and pembrolizumab is in the treatment of locally advanced or metastatic cutaneous sarcoma. T-VEC and pembrolizumab were both concomitantly administered on the first day of treatment and every 3 weeks thereafter. In an expansion cohort analysis of the study results, Kelly et al. stratified the study participants into three categories: undifferentiated pleomorphic sarcoma (UPS) and myxofibrosarcoma (MFS), epithelioid sarcoma (ES), and cutaneous angiosarcoma (AS). The investigators found that the optimal ORRs for each subgroup were 11% for UPS/MFS (*n* = 1/9, 95% CI: 0.0–0.48), 43% for AS (*n* = 3/7, 95% CI: 0.1–0.82), and 0% for ES (*n* = 0/3). Further, the best ORR was 71% for the AS subgroup (*n* = 5/7, 95% CI: 0.03–0.95), and the median PFS was 14.9 weeks for UPS/MFS and 54 weeks for AS. Only one study participant experienced one grade 3 TRAE, manifesting as immune-mediated hepatitis, and was withdrawn from the study [55]. This study demonstrates the safety and promising utility of T-VEC and pembrolizumab therapy in the treatment of advanced sarcomas.

### 3.2. RP-1 in NMSCs

In the IGNYTE (NCT03767348) trial, RP-1 was investigated as a therapeutic option for advanced solid tumors, including nonmelanoma HNSCCs. Specifically, RP-1 and RP-1 + nivolumab treatment was administered intratumorally every 2 weeks. The interim analysis yielded positive results, with notably high ORRs for BCC, MCC, and angiosarcoma of 25%, 75%, and 66%, respectively [62]. Included in the table above are two currently ongoing studies seeking to further characterize the impact of RP-1 on NMSCs.

The ongoing CERPASS clinical trial (NCT04050436) is looking to demonstrate the safety and efficacy of cemiplimab with or without concomitant RP-1 therapy in metastatic, unresectable SCC. The preliminary data analysis had very encouraging results: RP-1 in combination with cemiplimab increased the CRR compared to cemiplimab alone (38.1% vs. 25%, *p* = 0.040), and the completed response rate in the RP1-receiving group was 48.1% as opposed to 22.6% in the cemiplimab-only group. The ORR was comparable between both groups, with 52.5% for RP-1 plus cemiplimab vs. 51.4% for cemiplimab alone (*p* = 0.692). The RP-1-receiving group also displayed a longer duration of response time compared to cemiplimab alone. In terms of TRAEs, the RP-1-receiving group experienced predominantly flu-like symptoms, while the cemiplimab-only group experienced fatigue, nausea, pyrexia, pruritus, chills, diarrhea, and other low-grade adverse events [52]. The conclusion of this clinical trial, along with further studies on the efficacy of RP-1 in treating other NMSCs, will provide further clarity on the utility and safety of this new treatment option.

Additionally, the ARTACUS trial (NCT04349436) is currently investigating single-agent RP-1 in the treatment of advanced cutaneous malignancies in patients who have undergone solid organ transplants. In the report of the study’s interim results, it was found that single-agent RP-1 resulted in an ORR of 35%, with a CRR of 22%. Regarding TRAEs, the most common included fatigue (33%), chills (26%), and pyrexia (26%) [54]. The study results are hopeful for RP-1 as a therapeutic agent for advanced skin cancers, and the final results from the study will better elucidate if RP-1 as a monotherapy yields different results or efficacy from combination therapy with RP-1. Importantly, these encouraging findings would have particularly significant implications for recipients of solid organ transplants, as their high risk of organ rejection currently poses great limitations on their ability to receive and tolerate a number of immunotherapies. As mentioned previously, NMSCs are the most common malignancies among the recipients of solid organ transplants. Thus, the results from this trial could address a gap in treatment options for this distinct patient cohort. Therefore, it is important to continue investigating this approach in patients who have undergone solid organ transplants with NMSCs as well as investigate the potential benefit that RP-1 may hold in recipients of organ transplants with other solid tumor malignancies.

### 3.3. Other OV Usage in NMSCs

T3011 is another novel OV therapy with the same recombinant HSV-1 structure as T-VEC and RP-1. Administered as an IT injection, T3011 replicates selectively within tumor cells and spares healthy tissue like its T-VEC and RP-1 counterparts. It acts by incorporating the PD-1 antibody and IL-12, which enables it to uniquely act on the TME [63]. Currently, a phase II trial (NCT05602792) is investigating the use of T3011 IT injections as a monotherapy for advanced solid tumors. As mentioned previously, the interim results demonstrated an ORR of 11% (*n* = 6) and a DCR of 49% (*n* = 27) in 55 patients with HNSCC, sarcoma, melanoma, and breast cancer treated under RP2D who could be evaluated for their tumor response. Of the patients with HNSCC, 19 progressed, the median PFS was 3.9 months, and 12 patients who were evaluated post-dose had an ORR of 25% and a DCR of 50%. In terms of safety, the most common TRAEs were pyrexia, flu-like illness, elevated thyroid-stimulating hormone, proteinuria, facial edema, leukocytosis, elevated aspartate aminotransferase, and anemia [45]. In all, the interim results of this trial provide valuable insight into the utility of T3011 for the future treatment of advanced NMSCs, and a further analysis of the final outcome measure results will provide more insight into this therapeutic approach.

## 4. Future Directions and Conclusions

OVs have shown promising potency in skin cancer treatment based on the results of the clinical trials outlined. However, not all patients benefit equally from this treatment approach. As mentioned previously, OV responses vary greatly among different patients as they are dependent on the host’s immune response, treatment timepoint, type of malignancy, and the type of OVs administered. In addition, OVs have been studied in pre-treated patients with other therapeutics that could have altered the TME and, as a result, the clinical response to OVs [64]. Andtbacka et al. highlighted that patients treated with T-VEC as a first-line of therapy had greater DRR and ORR than those pre-treated [33], which highlights how pre-treatment could impact the clinical response to OVs. Further, the MASTERKEY-265 trial did not demonstrate any additional benefit from combining T-VEC with pembrolizumab over pembrolizumab alone. Therefore, more detailed studies on the determinants of efficacy of OVs in patients with skin cancers are needed.

Importantly, there remains a need for the identification of novel biomarkers for patient selection for OVs therapies. For instance, Nguyen et al. identified that mutations in the IFNγ-JAK-STAT pathway, which is an established biomarker for ICI resistance in melanoma, has increased melanoma cell lines’ susceptibility by 7- and 22-fold in two modified OVs, HSV1-dICP0 and vesicular stomatitis virus (VSV-Δ51), respectively [65]. Similarly, we previously reported a patient with refractory intracranial metastatic melanoma who acquired beta-2-microglobulin (B2M) mutation, which contributes to PD-1 resistance. Following the administration of T-VEC in combination with pembrolizumab and then Temozolomide (TMZ), the patient demonstrated an ongoing durable response [66]. Although it is unknown whether TMZ or T-VEC are responsible for the response in this patient, these results are imperative in understanding how OVs could fit the treatment algorithm against melanoma and other skin cancers and highlight the need to establish potential biomarkers for OV patient selection.

Furthermore, the usage of blockade inhibitors to enhance the action of OVs is another therapeutic route that requires further investigation. In a study using a murine PD-1–resistant melanoma model, treatment with MEK inhibitor (MEKi) therapy was found to significantly enhance the anti-tumor activity of T-VEC. Combining OVs and MEKi was found to also increase PD-L1 expression. Furthermore, combining anti-PD-1 therapy with T-VEC and MEKi showed a more augmented response, thus demonstrating preclinical promise in this therapeutic approach [67]. However, utilizing MEKi to further augment the action of OVs has not been studied extensively in the clinical setting. Thus, there continues to be a need to research this approach in skin cancer clinical studies that evaluate the potential synergic effect of combining checkpoint blockades with OVs.

In conclusion, OVs demonstrate great promise, as highlighted by the clinical results presented from 2022 to April 2024. Importantly, many new OV therapeutic approaches are currently being investigated, such as the use of the poliovirus and adenovirus as potential oncolytic agents, and advancements in genetic engineering have driven innovation in this field. However, there remains a need for a more personalized approach to patient selection for OVs. In addition, clinical investigation is needed to better understand the potential synergism between standard therapeutics, such as checkpoint blockades, and OVs. Nevertheless, OVs have progressed the landscape of skin cancer treatment, especially in patients experiencing disease progression while undergoing treatment with standard therapeutics. In addition, OVs have opened the door for a potential therapeutic route for patients who have undergone solid organ transplants with NMSCs who may not benefit from standard immunotherapies, as demonstrated by the ARTACUS trial (NCT04349436). Yet, future research is required to understand how this therapeutic approach can fit into the treatment algorithm against skin cancers and develop a more personalized approach.

## Figures and Tables

**Table 1:** Recent melanoma clinical trials and outcomes.

Authors, Study Name, NCT #	Trial Stage	Therapeutic Approach	N	Disease/Stage	Outcomes	Common TRAEs/Grade 3–4
Zijlker L. et al., 2023 NIVEC (NCT04330430) [38]	Phase II (Active, Not Recruiting)	Neoadjuvant T-VEC + Nivolumab for 8 weeks	24	Resectable stage IIIB-IVA melanoma	pCR in 65% of pts and a partial response in 9%.	Grade 3 TRAEs = 8%. No grade 4 TRAEs.
(NCT03842943)	Phase II (Recruiting)	T-VEC + pembrolizumab	28	Stage III	N/A	N/A
(NCT02965716)	Phase II (Active, Not Recruiting)	T-VEC + pembrolizumab	42	Stage III-IV	N/A	N/A
Gastman B. et al., 2022 MASTERKEY-115 (NCT04068181) [37]	Phase II (Completed)	T-VEC + pembrolizumab	72	Stage IIIB-IV	ORR ranged from 0 to 46.7% among different cohorts.	Most common TRAEs: pyrexia, fatigue, flu-like illness. Grade ≥ 3: 12.7%.
Schwarze J. et al., 2022 myDCTV (NCT03747744) [39]	Phase I (Status Unknown)	CD1c (BDCA-1) + myDC + T-VEC	18	Advanced/metastatic melanoma	15% developed a durable pCR, 8% partial unconfirmed response, and 15% mixed response.	Most common TRAEs: fatigue, fever, chills/flu-like symptoms.
Chmielowski B. et al., 2023 IGNYTE (NCT03767348) [40]	Phase II (Recruiting)	RP-1 vs. RP-1 + nivolumab	340	Advanced and/or refractory solid tumors, including melanoma	ORR was 37.4%, and CR was 18.7%.	Most common TRAEs: fatigue, chills, pyrexia, and nausea.
Barker C. et al., 2023 (NCT02819843) [41]	Phase II (Active, Not Recruiting)	T-VEC + Hypofractionated Radiotherapy vs. T-VEC alone	19	Solid tumors, including melanoma	Composite response rate in T-VEC = 7% and T-VEC+RT = 22%. (Trial was closed early due to slow accrual)	Most common TRAEs was fever.
Yamazaki N. et al., 2022 (NCT03064763) [42]	Phase I (Completed)	T-VEC	18	Stage IIIB-IV	11.1% had a durable partial response ≥ 6 months.	Most common TRAEs were pyrexia, malaise, chills, decreased appetite, pruritus, and skin ulcers.
Dummer R. et al., 2023 (NCT02211131) [43]	Phase II (Completed)	Neoadjuvant T-VEC for 6 doses + surgical resection vs. immediate surgical resection	150	Resectable stage IIIB- IVM1a	For group 1 (T-VEC then surgery) and group 2 (immediate surgery), respectively, 5-year Kaplan–Meier plots showed 22.3% and 15.2% for RFS; 43.7% and 27.4% for EFS; and 77.3% and 62.7% for OS. HRs for local RFS, regional RFS, and DMFS were 0.82 0.81, and 0.73, respectively.	No new safety signals were reported.
LUMINOS-102 (NCT04577807)	Phase II (Active, Not Recruiting)	PVSRIPO vs. PVSRIPO + anti-PD-1	56	Advanced melanoma refractory to anti-PD-1	N/A	N/A
Wei X. et al., 2022 (NCT04197882) [44]	Phase IB (Active, Not Recruiting)	Neoadjuvant OrienX010 + toripalimab for 8–12 weeks and Adjuvant toripalimab for a year	30	Resectable stage IIIB-IV (M1a) acral melanoma.	Radiological ORR = 36.7% and pathological ORR = 77.8%. CRR = 3.3%. 1-year RFS = 80%.	Grade 3 TRAEs= 17% (soft tissue infections, transaminitis, peripheral neuropathy, and neutropenia).
(NCT06216938)	Phase I (Recruiting)	RP-1	25	Primary melanoma	N/A	N/A
(NCT05961111)	Phase I (Recruiting)	R130 OV	20	Advanced solid tumors, including melanoma	N/A	N/A
(NCT05868707)	Phase III (Recruiting)	OH2 vs. Salvage chemotherapy or best supportive care	340	Unresectable or metastatic melanoma	N/A	N/A
Ji, D. et al., 2023 (NCT05602792) [45]	Phase I/IIa (Recruiting)	T3011	233	Advanced solid tumors, including melanoma	The confirmed ORR was 11%, the DCR was 49% in 55 pts including HNSCC, sarcoma, melanoma, breast cancer, etc., treated under RP2D evaluable for tumor response. The median PFS was 2.8 months in all 76 pts receiving T3011.	Both TRAEs of ≥G3 and treatment discontinuation in 2.2% of pts. The most frequently reported TRAEs were pyrexia (21.1%), influenza-like illness (8.9%), increased TSH (6.7%), proteinuria (6.7%), facial edema, white blood cell count decreased, AST increased, and anemia (5.6%).
(NCT04725331)	Phase I/II (Recruiting)	BT-001 alone or in combination with pembrolizumab	48	Advanced solid tumors, including melanoma	N/A	N/A
(NCT05076760)	Phase I (Recruiting)	MEM-288 alone or in combination with nivolumab	61	Advanced solid tumors, including melanoma	N/A	N/A
(NCT06265025)	Phase I/II (Recruiting)	GM103 alone and in combination with pembrolizumab	125	Locally advanced, unresectable, refractory, and/or metastatic solid tumors, including melanoma	N/A	N/A
Santos, J. et al., 2023 AVENTIL (NCT05222932) [46]	Phase I (Recruiting)	TILT-123 and avelumab	15	Solid tumors refractory to or progressing after anti-PD-1, including melanoma	N/A	N/A
Monberg T. J. et al., 2023 TUNINTIL (NCT04217473) [47]	Phase I (Active, Not Recruiting)	TILT-123 with tumor-infiltrating lymphocytes	17	Advanced melanoma	Disease control rate of 38.0%.	Most common AEs from TILT-123 were fever and pain at injection site.
(NCT06171178)	Phase I (Recruiting)	ASP1012	229	Advanced/metastatic solid tumors, including melanoma	N/A	N/A
(NCT04616443)	Phase I/II (Recruiting)	OH2 in combination with HX008	60	Advanced melanoma	N/A	N/A

Abbreviations: N/A = not available; TRAE = treatment-related adverse events; pCR = pathological complete response; ORR = objective response rate; pts = patients; CRR = complete response rate; RFS = recurrence-free survival; CR = complete response; EFS = event-free survival; OS = overall survival; HRs = hazard ratios; DMFS = distant metastasis-free survival; and AEs =adverse events.

## Data Availability

Data sharing is not applicable.
